# Systemic Inflammatory Indices—Systemic Immune-Inflammation Index (SII) and the Systemic Inflammation Response Index (SIRI)—As Potential Rule-Out Biomarkers for Invasive Cervical Carcinoma

**DOI:** 10.3390/ijms27010435

**Published:** 2025-12-31

**Authors:** Márton Keszthelyi, Réka Eszter Sziva, Zsófia Havrán, Verita Szabó, Noémi Kalas, Lotti Lőczi, Barbara Sebők, Petra Merkely, Nándor Ács, Szabolcs Várbíró, Balázs Lintner, Richárd Tóth

**Affiliations:** 1 Department of Obstetrics and Gynecology, Semmelweis University, Üllői Road 78/A, 1082 Budapest, Hungary; keszthelyi.marton@semmelweis.hu (M.K.); keszthelyi.lotti.lucia@semmelweis.hu (L.L.); merkely.petra@gmail.com (P.M.); acs.nandor@semmelweis.hu (N.Á.); lintner.balazs.zoltan@semmelweis.hu (B.L.); toth.richard@semmelweis.hu (R.T.); 2 Faculty of Medicine, Semmelweis University, Üllői Road 26, 1085 Budapest, Hungary; zsofi.havran@gmail.com (Z.H.); szabo.verita@gmail.com (V.S.); kalasnoemi@gmail.com (N.K.); 3 Workgroup of Research Management, Doctoral School, Semmelweis University, Üllői Road 26, 1085 Budapest, Hungary; sebok.barbara23@gmail.com (B.S.); varbiroszabolcs@gmail.com (S.V.); 4 Department of Obstetrics and Gynecology, University of Szeged, Semmelweis Street 1, 6725 Szeged, Hungary

**Keywords:** SII, SIRI, cervical cancer, complete blood count, HPV

## Abstract

Cervical cancer, primarily caused by high-risk Human Papilloma Virus (HPV), remains a global health concern. Prognostic biomarkers reflecting systemic inflammation and immune response—the Systemic Immune-Inflammation Index (SII) and the Systemic Inflammation Response Index (SIRI)—have recently attracted interest for their potential predictive value in cervical cancer. We conducted a retrospective observational study including 344 patients who underwent loop electrosurgical excision of cervical intraepithelial neoplasia at Semmelweis University, Budapest, Hungary, between 2021 and 2024. Demographic, cytologic, histologic, and laboratory data were collected, and SII and SIRI were calculated. Statistical analyses, including Receiver Operating Characteristic (ROC) analyses, were performed. Higher SII and SIRI values were significantly associated with higher-grade lesions and invasive carcinoma. ROC analyses indicated good discriminatory performance, with negative predictive values of 96–100%, suggesting potential utility in ruling out malignant transformation. SII and SIRI are simple, cost-effective, and minimally invasive biomarkers that correlate with lesion severity in cervical disease. Their high negative predictive value supports a potential role as complementary rule-out tools in diagnostic evaluation. Further prospective studies are needed to validate these findings and to define clinically meaningful cut-off values for routine use.

## 1. Introduction

Cervical cancer remains a global health concern, with incidence increasing in developing countries, largely due to high-risk Human Papilloma Virus (HPV) infections [1,2]. HPV, a common sexually transmitted virus, infects the epithelium of the cervix as well as other anogenital and oropharyngeal sites [3,4]. Cervical cancer usually develops gradually from precancerous lesions called cervical intraepithelial neoplasia (CIN), which are classified into three grades: CIN I (mild dysplasia), CIN II (moderate dysplasia), and CIN III (severe dysplasia or carcinoma in situ) [5]. Despite progress in screening methods, CIN is often diagnosed at advanced stages due to inadequate early detection and inconsistent follow-up [6]. The loop electrosurgical excision procedure (LEEP) is widely used for the definitive treatment of advanced CIN, removing the abnormal cervical tissue [7,8].

The Systemic Immune Inflammation Index (SII) and the Systemic Inflammation Response Index (SIRI) have recently attracted attention in cervical cancer research as potential prognostic biomarkers reflecting the interaction between host immune function and systemic inflammation. Both indices are derived from routine blood count parameters, making them cost-effective, accessible, and non-invasive tools for clinical practice.

The SII, calculated as:

$$SII=\;\frac{Platelet\;count\left(\;\frac{G}{L}\right)*\;Neutrophil\;granulocyte\;count(\frac{G}{L})}{Lymphocyte\;count\;(\frac{G}{L})},$$

reflects the balance between pro-tumor inflammatory cells (neutrophils and platelets) and the antitumor immune activity of lymphocytes. Elevated SII values have been consistently linked to advanced stage, higher tumor burden, lymph node involvement, and poorer overall survival [9]. Notably, SII has demonstrated superior prognostic value compared with individual ratios such as the neutrophil-to-lymphocyte ratio (NLR) and platelet-to-lymphocyte ratio (PLR), as it integrates multiple hematologic markers into a single index [9,10].

The SIRI, is calculated as:

$$SIRI=\;\frac{Neutrophil\;granulocyte\;count\left(\;\frac{G}{L}\right)*\;Monocyte\;count(\frac{G}{L})}{Lymphocyte\;count\;(\frac{G}{L})}$$

and includes monocytes, which are precursors of tumor-associated macrophages involved in angiogenesis, tumor progression, and immune evasion [11,12,13]. Including monocytes provides a broader measure of tumor-related inflammation, which may contribute to SIRI’s prognostic value in various cancers, including cervical cancer. Higher SIRI levels have been associated with advanced stage, lymph node involvement, treatment resistance, and reduced overall survival [14,15]. These indices highlight the prognostic relevance of systemic inflammation in precancerous cervical lesions and cervical cancer. While both SII and SIRI outperform traditional single hematologic ratios, SIRI may offer additional value by capturing monocyte activity and its downstream contribution to tumor-promoting immune mechanisms.

The present study evaluates the prognostic relevance of the SII and SIRI indices for disease progression among patients undergoing LEEP for cervical intraepithelial neoplasia. Furthermore, the study seeks to determine clinically meaningful cut-off values for both indices to enhance the prediction of underlying malignancy.

## 2. Results

### 2.1. Sociodemographic Characteristics

The final study population comprised 344 patients. Median age was 40 years, median body weight was 63 kg, and median BMI was 22.77 kg/m^2^. (last two parameters are for 339 cases). Smoking was reported by 56 participants (16.3%), while 282 (82.0%) were non-smokers; data were missing in 6 cases (1.7%).

Alcohol use was reported in only 1 case (0.3%), denied in 335 (97.4%), and missing in 8 (2.3%). Cytological distribution was G1: 8 (2.3%), G2: 66 (19.2%), G3: 252 (73.3%), and G4: 18 (5.2%). Hr-HPV positivity was found in 196 cases (57.0%), negativity in 8 (2.3%) patients, and data was unavailable for 140 (40.7%) cases. Among hr-HPV-positive cases, specific types were identified in 54 patients (27.5%): a single hr-HPV type in 39 (72.2%), two types in 10 (18.5%), and three types in 5 (9.3%) patients. Median SII was 516.5 G/L (IQR: 358.8–701.6), and median SIRI was 0.879 G/L (IQR: 0.611–1.24) (Table 1).

### 2.2. Distribution of Identified hr-HPV Types

Among the 54 hr-HPV-positive samples, 39 contained a single hr-HPV type, most commonly HPV16 and HPV31. Dual infections were observed in 10 cases, most frequently the HPV16+HPV18 combination (*n* = 3), while 5 cases showed triple hr-HPV infections. (Supplementary Materials S1§, Diagrams S1–S3).

### 2.3. Relationship Between Indices and Cytological Results

- Systemic Immune-Inflammation Index/SII

**Figure 1:**
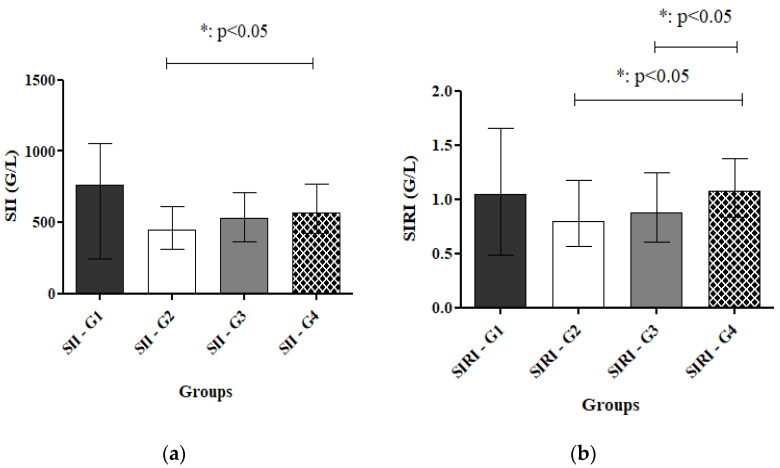
(**a**) Distribution of cervical cancer test screening results by G1-G4 categories for SII. The Systemic Immune-inflammation index is significantly higher in the Grade 4 group compared to the Grade 2 group. *: *p* < 0.05 for G2 vs. G4. (**b**) Distribution of cervical cancer test screening results by G1–G4 categories for SIRI. The Systemic Inflammation Response Index is significantly higher in the Grade 4 group compared to the Grade 2 and Grade 3 groups. *: *p* < 0.05 for G2 vs. G4 and G3 vs. G4. Abbreviations: SII: Systemic Immune-inflammation Index. SIRI: Systemic Inflammation Response Index. G1–4: Grade 1–4. Mann–Whitney-U test: MWU. All data are presented in Median [IQR].

The median SII values varied across cervical cancer screening grades. Grade 1 cases showed the highest median SII (764), although the small sample size (*n* = 8) was associated with wide variability (IQR: 246.8–1052). In contrast, Grade 2 patients demonstrated the lowest median SII (447.9, IQR: 310.3–609.0). Grade 3, representing the largest subgroup (*n* = 252), had a median SII of 530.2 (IQR: 364.7–711.1), while Grade 4 patients (*n* = 18) exhibited a slightly higher median SII (564.1, IQR: 428.7–773.5). Overall, the lowest SII levels were observed in Grade 2, whereas Grade 3 and 4 patients displayed moderately elevated but comparable median values (Supplementary Materials S2§, Tables S1 and S2). Statistical analysis of SII values across cytological grades showed a significant difference exclusively between Grade 2 and Grade 4 (*p* = 0.0443), where Grade 4 was associated with higher SII levels. No other pairwise comparisons were statistically significant, although the Grade 2 vs. Grade 3 comparison showed a trend toward significance (*p* = 0.0586) (Figure 1a).

- Systemic Inflammation Response Index/SIRI

SIRI values varied across the groups of patients, with minimum and maximum values of 0.0281 and 5.557, respectively. When stratified by grade, median SIRI values were 1.051 for Grade 1, 0.7977 for Grade 2, 0.8753 for Grade 3, and 1.074 for Grade 4. Interquartile ranges overlapped across grades, especially between Grades 2 and 3, indicating moderate within-group variability. Grade 3 showed the widest range, encompassing both the cohort minimum and maximum. Overall, SIRI values tended to increase with lesion grade, though considerable intra-grade variability remained (Supplementary Materials S3§, Tables S3 and S4). Pairwise comparisons of SIRI values across grades showed no significant differences between G1 and G2 (*p* = 0.3794), G1 and G3 (*p* = 0.6077), or G1 and G4. Similarly, the comparison between G2 and G3 did not reach statistical significance. However, SIRI values were significantly higher in the G4 group compared to G2 (*p* = 0.0237) and compared to G3 (*p* = 0.0434) (Figure 1b). These results indicate that elevated SIRI is associated with the highest-grade lesions, while differences among lower-grade lesions were not statistically significant.

### 2.4. Relationship Between Indices and Histological Results After LEEP-Conization

- Systemic Immune-Inflammation Index/SII

Among the population, overall median SII values varied according to histological grade. Median SII was 510.3 in G1 (*n* = 72), with an interquartile range (IQR) of 315.8–675.4, ranging from 193.8 to 1928. In G2 (*n* = 30), the median was 448.0 (IQR 356.4–582.9; range 161.8–1322), while in G3 (*n* = 219), the median SII was 506.0 (IQR 358.2–694.5; range 121.3–1962). The highest median SII was observed in G4 (*n* = 23) at 767.5, with an IQR of 550.0–1097 and a range of 217.9–1897. Overall, IQRs overlapped across most grades, particularly between G1 and G3, reflecting moderate variability within these groups. Notably, the highest median and upper-range values were observed in G4, indicating a tendency toward elevated SII in the most severe histological category (Supplementary Materials S4§, Tables S5 and S6).

Pairwise comparisons of SII values with the MWU test showed no significant differences between G1 and G2 (*p* = 0.456), G1 and G3 (*p* = 0.8686), or G2 and G3 (*p* = 0.2887).

**Figure 2:**
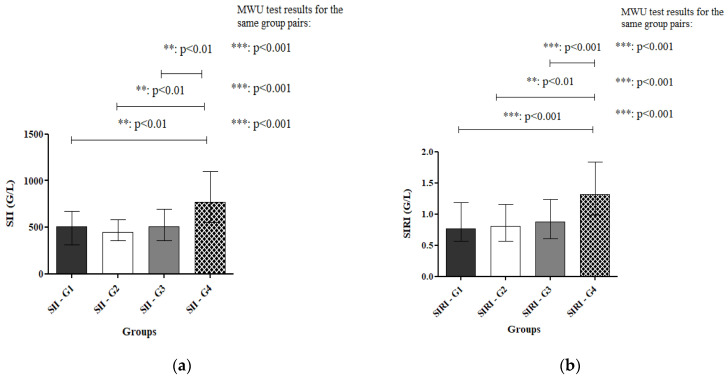
(**a**) Distribution of histological results by G1-G4 categories for SII. The Systemic Immune Inflammation index is significantly higher in the Grade 4 group compared to Grade 1, Grade 2 and Grade 3 groups. Kruskal–Wallis test: **: *p* < 0.01 for G1 vs. G4, G2 vs. G4 and G3 vs. G4. Mann–Whitney-U test: ***: *p* < 0.001 for G1 vs. G4, G2 vs. G4 and G3 vs. G4. (**b**) Distribution of histological results by G1–G4 categories for SIRI. The Systemic Inflammation Response Index is significantly higher in the Grade 4 group compared to Grade 1, Grade 2 and Grade 3 groups. Kruskal–Wallis test: ***: *p* < 0.001 for G1 vs. G4 and G3 vs. G4, **: *p* < 0.01 for G2 vs. G4. Mann–Whitney-U test: ***: *p* < 0.001 for G1 vs. G4, G2 vs. G4 and G3 vs. G4. Abbreviations: SII: Systemic Immune Inflammation Index. SIRI: Systemic Inflammation Response Index. G1–4: Grade 1–4. Kruskal–Wallis test and Dunn’s post hoc test, Mann–Whitney-U test (MWU). Data are presented in Median [IQR].

In contrast, SII values were significantly higher in the G4 group compared with all lower-grade groups: G1 vs. G4 (*p* = 0.0007), G2 vs. G4 (*p* = 0.0006), and G3 vs. G4 (*p* = 0.0003). These results indicate that elevated SII is associated with the highest-grade lesions, while differences among lower-grade lesions did not reach statistical significance (Figure 2a).

- Systemic Inflammation Response Index/SIRI

Median SIRI values were as follows: 0.7657 in G1 (*n* = 72, IQR 0.5702–1.187; range 0.3367–3.509); 0.8027 in G2 (*n* = 30, IQR 0.5702–1.162; range 0.2841–2.436); 0.8770 in G3 (*n* = 219, IQR 0.6103–1.234; range 0.0281–4.279), and 1.32 in G4 (*n* = 23, IQR 0.9822–1.836; range 0.6802–5.557). IQRs overlapped across lower grades, particularly between G1 and G2, indicating moderate intra-grade variability. The highest median and upper-range values were observed in G4, reflecting a tendency toward elevated SIRI in the most severe histological category, while substantial variability persisted within each grade (Supplementary Materials S5§, Tables S7 and S8). Pairwise comparisons of SIRI by histological grades using the Mann–Whitney-U test indicated that there were no significant differences between G1 and G2 (*p* = 0.7053), G1 and G3 (*p* = 0.3690), or G2 and G3 (*p* = 0.3574). In contrast, SIRI values were significantly higher in G4 compared with all lower-grade groups: G1 vs. G4 (*p* = 0.0001), G2 vs. G4 (*p* = 0.0003), and G3 vs. G4 (*p* < 0.0001). These results indicate that elevated SIRI is associated with the highest-grade lesions, while differences among lower-grade lesions did not reach statistical significance (Figure 2b).

### 2.5. Results of the ROC Analyses

#### 2.5.1. ROC-Analysis for Cervical Cancer Test Screening and the Indices

- Systemic Immune-Inflammation Index/SII

**Figure 3:**
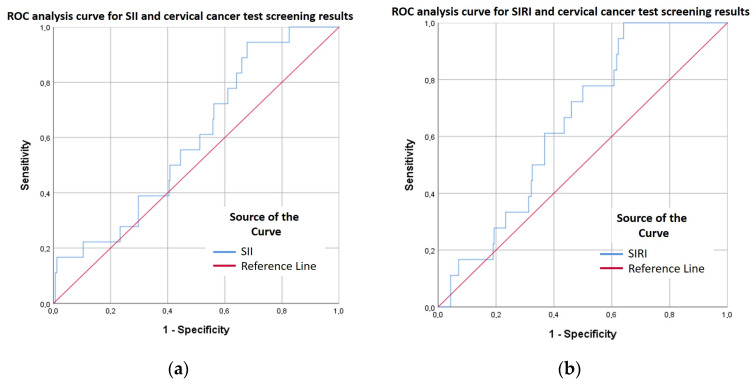
(**a**) ROC analysis curve for SII and cervical cancer test screening results. X: 1-Specificity, Y: Sensitivity. (**b**) ROC analysis curve for SIRI and cervical cancer test screening results. X: 1-Specificity, Y: Sensitivity. Blue line indicates SII (**a**)/SIRI (**b**), while red line represents reference lines.

Based on the ROC curve, SII demonstrated moderate discriminatory ability, with an AUC of 0.597. The optimal cut-off value determined by the Youden index was SII > 382.40, yielding a sensitivity of 94.4% and a specificity of 32.2%. The Closest Top Left method suggested a slightly higher cut-off (SII > 461.34), associated with a sensitivity of 72.2% and a specificity of 43.9%. To better assess clinical applicability, we also calculated the positive predictive value (PPV) and negative predictive value (NPV) for each threshold. For the Youden-based cut-off, PPV was 7.1% and NPV was 99.1%. Using the Closest Top Left cut-off, PPV and NPV were 6.6% and 96.6%, respectively. These results indicate that although the SII has limited value for confirming malignant cases (low PPV), it may serve as a useful exclusion tool due to its high negative predictive value (Figure 3a, Supplementary Materials S6§, Table S9).

- Systemic Inflammation Response Index/SIRI

The diagnostic performance of the SIRI was moderate in identifying malignant cytological cases (AUC = 0.647). The cut-off determined by the Youden index (SIRI ≥ 0.691) achieved excellent sensitivity (100%) but was accompanied by low specificity (35.9%) and a low positive predictive value (7.9%). The Closest Top Left method yielded a more conservative cut-off (SIRI ≥ 1.038), resulting in more balanced sensitivity (61.1%) and specificity (63.2%). These findings suggest that SIRI has limited utility for confirming malignant cases (low PPV), but given its high negative predictive value (96–100%), it may be clinically useful as a rule-out tool (Figure 3b, Supplementary Materials S6§, Table S10).

#### 2.5.2. ROC-Analysis for Histological Examination

- Systemic Immune-Inflammation Index/SII

**Figure 4:**
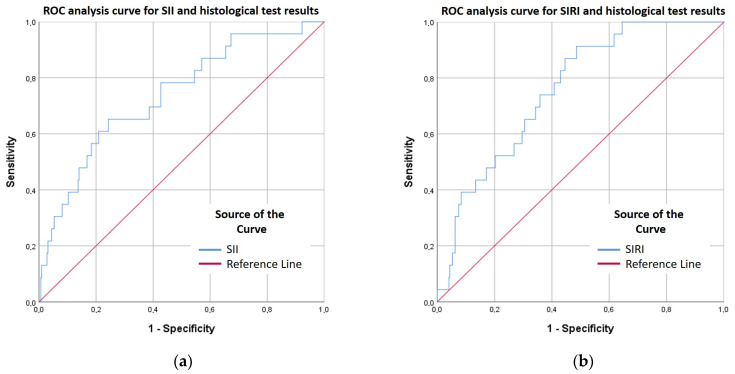
(**a**) ROC analysis curve for SII and histological test results. X: 1-Specificity, Y: Sensitivity. (**b**) ROC analysis curve for SIRI and histological test results. X: 1-Specificity, Y: Sensitivity. Blue line indicates SII (**a**)/SIRI (**b**), while red line represents reference lines.

The SII showed good discriminatory performance in identifying histologically confirmed malignant cases (AUC = 0.737). Both the Youden index and the Closest Top Left method identified the same optimal cut-off (SII ≥ 681.74), providing 65.2% sensitivity and 75.7% specificity. Although the PPV remained low (16.1%), the NPV was high (96.8%). Indicating that SII is more clinically applicable as an exclusion marker rather than a confirmatory diagnostic tool (Figure 4a, Supplementary Materials S7§, Table S11).

- Systemic Inflammation Response Index/SIRI

At the cut-off of 0.915 determined by the Youden index, the model demonstrated high sensitivity, meaning it reliably identified malignant cases, but also produced a higher number of false positives. The Closest Top Left method likewise provided a favorable balance between sensitivity and specificity. The PPV was low, indicating that only a small proportion of positive predictions corresponded to true malignant cases. Conversely, the NPV was high, supporting the use of the model as a rule-out tool, although insufficient for a standalone diagnostic application. (Figure 4b, Supplementary Materials S7§, Table S12).

### 2.6. Multivariable Logistic Regression

To evaluate whether SII and SIRI were independently associated with invasive cervical cancer, multivariable logistic regression analyses were performed using histologically confirmed invasive disease (Grade IV) as the dependent variable.

In the primary model including SII, age, and smoking status (N = 338), higher SII values were independently associated with invasive cancer (OR = 1.002 per unit increase, *p* < 0.001). Age was also a significant predictor (OR = 1.061 per year, *p* = 0.005), while smoking status was not significantly associated with invasive disease. The overall model showed good fit (Nagelkerke R^2^ = 0.176; omnibus χ^2^ = 24.13, *p* < 0.001). In an extended model additionally adjusted for BMI (N = 335), the association between SII and invasive cancer remained significant (OR = 1.002, *p* < 0.001), as did age (OR = 1.068, *p* = 0.003), while BMI and smoking status were not significant predictors. Model fit was similar (Nagelkerke R^2^ = 0.183).

A secondary multivariable model including SIRI instead of SII demonstrated that higher SIRI values were independently associated with invasive disease (OR = 2.308, *p* < 0.001), together with age (OR = 1.058, *p* = 0.005), whereas smoking status was not significant (Nagelkerke R^2^ = 0.140; omnibus χ^2^ = 19.02, *p* < 0.001). When BMI was additionally included in the SIRI-based model (N = 335), the association between SIRI and invasive disease remained statistically significant, while BMI did not emerge as an independent predictor, and overall model performance did not materially improve (Supplementary Materials S8§).

## 3. Discussion

In a retrospective cohort of women undergoing LEEP, we assessed the clinical relevance of the SII and SIRI indices in relation to cytological and histological outcomes. Both indices were significantly elevated in patients with high-grade cervical lesions and invasive cancer compared to those with low-grade lesions or negative findings. In ROC analyses, SII and SIRI showed good discriminatory performance, with relatively high negative predictive values suggesting a possible role as rule-out markers in the diagnostic evaluation of cervical lesions.

These indices reflect the balance between systemic inflammation and immune response. In malignancies, elevated levels may mirror a shift toward a tumor-supportive microenvironment associated with inflammation, angiogenesis, and reduced antitumor immune activity [16]. Retrospective studies in hematologic malignancies have linked higher SII values to poorer survival [17]. In colorectal cancer, Menyhart et al. demonstrated that both elevated SII and SIRI are linked to worse survival outcomes [18]. A meta-analysis confirmed the adverse prognostic role of SII [19], and pooled data across solid tumors showed that higher SIRI correlates with unfavorable prognosis [20].

These findings highlight the potential prognostic relevance of systemic inflammation indices across different malignancies and support their evaluation in cervical cancer. Chao et al. previously demonstrated that elevated SIRI is independently associated with poor survival and lymph node involvement in cervical cancer, while Huang et al. reported comparable prognostic significance for SII [9,11]. Our findings build on these observations by showing that both markers are associated not only with histologically confirmed malignancy but also with cytological screening results. In our study, SII and SIIR clearly identified invasive cervical cancer that was diagnosed by histology-based analysis, with both SII and SIRI values being significantly elevated in the Grade 4 group compared with lower-grade groups, supporting their possible role as markers of disease severity. SIRI demonstrated an AUC of 0.757 and a negative predictive value of up to 98.3%, indicating strong sensitivity for malignancy, although it was less reliable for differentiating between G2 and G3 lesions. Pairwise comparisons revealed a statistically significant difference only between Grade 2 and Grade 4. The limited separation between G1 and higher-grade tumors likely reflects the small number of G1 cases in the cohort.

These observations are further supported by previous literature. A meta-analysis including 1271 cervical cancer patients demonstrated that elevated SIRI was significantly associated with advanced FIGO stage and high-grade histology, highlighting the prognostic relevance of SIRI across disease severity [20]. Similarly, Liana et al. reported significant elevations in both SII and SIRI in stage III–IV cervical cancer compared with stage I–II, reflecting hematological shifts associated with cancer progression [21]. Collectively, these results underscore the potential utility of SII and SIRI as integrative, peripheral blood-based biomarkers for stratifying cervical cancer severity, particularly in identifying invasive disease.

Our histology-based analysis reinforced these observations. SII values were markedly elevated in Grade 4 compared with all lower-grade groups, supporting its role as a marker of invasive disease. SIRI, likewise, showed progressive increases across histological categories, with the highest median values in invasive cancer. Significant differences were consistently observed between Grade 4 and each of the lower-grade groups, underscoring the robustness of SIRI in reflecting histological severity. Together, these findings suggest that while both indices have diagnostic relevance, SIRI may offer superior discriminatory power across cytological and histological assessments, particularly for identifying invasive cervical cancer.

Conventional inflammatory markers, such as NLR and PLR, have been associated with cervical tumor development and may reflect systemic inflammation and immune dysregulation. Specifically, PLR may better capture concurrent thrombopoietic activation and lymphopenia, integrating both pro-tumor inflammatory and impaired antitumor immune responses. Elevated platelet counts are frequently linked to tumor angiogenesis and progression, whereas lymphocytopenia indicates compromised antitumor immunity [7,22]. While these traditional ratios provide valuable insight, they often fail to fully encompass the complex interplay between systemic inflammation and tumor progression. In contrast, SII and SIRI combine multiple hematologic parameters into a single index, offering a more comprehensive reflection of pro-tumor inflammation and host immune competence. Recent studies have demonstrated that SII and SIRI provide superior predictive accuracy for CIN and cervical cancer progression compared to NLR, PLR, and LMR [23]. Notably, SIRI alone has shown a high NPV for excluding CIN recurrence within two years (90.2%) [24]. Similarly, Han et al. reported that high NLR and PLR were significantly associated with poor prognosis in cervical cancer, further supporting the prognostic utility of systemic inflammatory markers [25].

In our histology-based analyses, SII achieved an NPV of 96.8%, whereas SIRI demonstrated slightly superior discriminatory capacity with an NPV of 98.3%. Although positive predictive values were limited (SII: 16.1%, SIRI: 12.3%), the very high NPVs indicate that these biomarkers are particularly reliable for excluding malignancy in patients with ambiguous cytological results, potentially reducing unnecessary invasive procedures. These findings highlight the enhanced utility of SII and SIRI as integrative, peripheral-blood-test-based biomarkers for cervical cancer risk stratification [26]. Although SII and SIRI showed relatively high negative predictive values, these results should be considered in the context of the low prevalence of invasive disease in the study population. The moderate AUC values suggest that these indices have limited discriminative ability and are better viewed as complementary rather than definitive diagnostic tools. Future prospective multicenter studies with larger sample sizes are needed to validate our findings, assess their clinical utility, and explore how SII and SIRI could be integrated into existing cervical cancer screening and management strategies.

Additionally, combining these indices with HPV genotyping, molecular biomarkers, or radiological features may further enhance their predictive accuracy. SII and SIRI have already emerged as promising complementary markers in cervical disease. They are not intended as standalone diagnostic tools, but rather provide adjunctive information to assess disease severity and progression. Liana et al.’s cross-sectional study demonstrated significant differences in SII and SIRI between stage III and IV cervical cancer and stages II and I, reflecting shifts in routine hematological parameters with advancing disease [21]. In line with this, a recent meta-analysis has shown that high SII and SIRI levels are associated with poorer overall survival in cervical cancer, further supporting their prognostic relevance [27]. These findings support the complementary value of systemic inflammatory indices alongside conventional diagnostic modalities. Determining optimal cut-off values for these indices is essential for translating these biomarkers into clinical practice, as they define thresholds that distinguish patients at higher risk of recurrence from those with a low likelihood of disease.

It is also valuable to consider the immunological and molecular mechanisms underlying HPV-driven carcinogenesis and the tumor microenvironment. HPV-driven carcinogenesis involves not only genetic and epigenetic changes but also an immunologically influenced trajectory, in which viral oncogene expression (E6/E7) can promote local immune evasion and chronic inflammation [28]. Mechanistically, HPV-associated tumors may suppress innate immune sensing and interferon signaling, and are often associated with upregulation of immune checkpoints, such as PD-L1 on tumor and infiltrating immune cells, contributing to T-cell dysfunction [29]. In parallel, HPV-related lesions exhibit cytokine and chemokine programs (including IL-6/IL-8 and other myeloid-recruiting signals) that support expansion and recruitment of suppressive myeloid compartments, including tumor-associated macrophages (TAMs) and tumor-associated neutrophils (TANs) [30]. Within this framework, elevated SII and SIRI likely reflect a systemic pro-tumor immune–inflammatory state characterized by neutrophil and monocyte predominance with relative lymphopenia, indicating impaired antitumor immunity. Neutrophils and platelets contribute to angiogenesis, immune evasion, and tumor progression, while circulating monocytes serve as precursors of tumor-associated macrophages that promote immune suppression and extracellular-matrix remodeling within the HPV-driven tumor microenvironment [31,32,33]. Similarly, an elevated SIRI is biologically consistent with HPV-associated tumor-microenvironment remodeling, where circulating monocytes serve as precursors for TAM populations that sustain immune suppression (e.g., IL-10/TGF-β signaling, PD-L1 expression, T_reg_ recruitment) and extracellular-matrix remodeling—features repeatedly implicated in cervical cancer progression [34]. In this context, SIRI may act as an accessible peripheral surrogate for a “myeloid-high/lymphocyte-low” immune state that aligns with invasive transformation, which could partly explain its stronger separation of Grade 4 disease in our cohort.

In summary, our study supports the prognostic and diagnostic relevance of Systemic Immune-Inflammation and System Inflammation Response Indices (SII and SIRI) in women undergoing conization for cervical intraepithelial neoplasia. Both indices were significantly associated with disease severity. They demonstrated high negative predictive values, underscoring their potential role as simple, cost-effective, and non-invasive tools to complement conventional screening methods in cervical cancer care. Further prospective, multicenter studies are warranted to validate these biomarkers and to define clinically actionable cut-off values for integration into routine cervical cancer risk stratification.

Limitations: This study has several limitations, including its retrospective, single-center design, the absence of established cut-off values, and the inclusion of only patients undergoing LEEP conization, which may introduce selection bias and the potential influence of unmeasured confounders. Another important limitation of the present study is the high proportion of missing hr-HPV data. Due to the retrospective nature of the cohort and changes in clinical documentation over time, hr-HPV status was not consistently available, particularly in earlier cases, which limited our ability to adjust for this established risk factor. In addition, the imbalance between invasive and non-invasive diagnostic groups reflects the real-world prevalence of invasive cervical cancer in a conization cohort but may have influenced measures of diagnostic performance, including predictive values. Finally, although SII and SIRI demonstrated high negative predictive values, these findings should be interpreted cautiously given the low prevalence of invasive disease in the study population.

## 4. Materials and Methods

### 4.1. Patient Selection, Inclusion, and Exclusion Criteria

**Figure 5:**
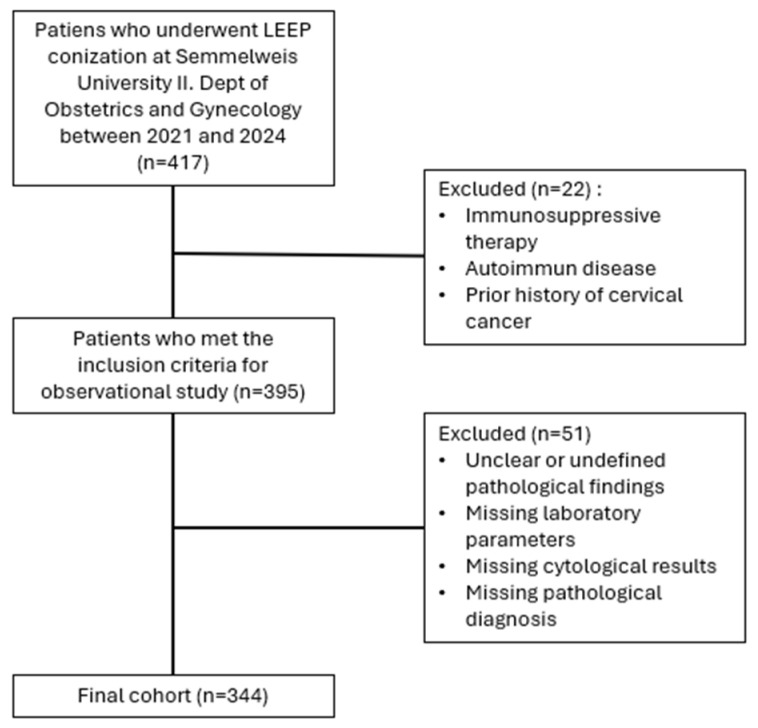
Study flowchart and patient selection/exclusion criteria. *n* = sample size.

This retrospective observational study evaluated a cohort of 417 patients within the framework of the SCOPE Study (Semmelweis University Conization and Inflammation Outcomes with Predictive Evaluation). Medical records from 2021 to 2024 were extensively reviewed to construct a comprehensive dataset, which included patient sociodemographic characteristics, gynecological history (cervical cancer screening results, HPV status with emphasis on high-risk HPV (hr-HPV), and histological outcomes of conizations), and preoperative laboratory parameters. Hr-HPV refers to a group of oncogenic HPV genotypes that are associated with a high risk of progression to high-grade cervical intraepithelial neoplasia and cervical cancer. The most common high-risk types include HPV 16 and 18, as well as types 31, 33, 35, 45, 52, and 58. Inclusion criteria were limited to adult patients aged 18 years or older who underwent conization for the management of CIN and had complete clinical, cytological, histological, and laboratory data available. Patients were excluded if they had unclear or undefined or non-neoplastic pathological findings (e.g., cervical polyps, coilocytotic atypia), missing or incomplete laboratory parameters, missing or incomplete cytological results, or missing final pathological diagnosis. In addition, patients’ medical histories were reviewed to identify systemic comorbid conditions that could influence systemic inflammatory markers, including autoimmune and/or immunological disorders, a history of immunosuppressive therapy, and a prior history of cervical cancer. After applying the exclusion criteria, data from 344 patients were available for final analysis. (Figure 5). The study was conducted in accordance with the Declaration of Helsinki and approved by the Institutional Review Board (or Ethics Committee) of Semmelweis University (SE RKEB: 195/2024). Informed consent was obtained from all subjects involved in the study.

All data were entered into a dedicated SCOPE Study database and underwent rigorous validation for accuracy, consistency, and outliers using box plots. Missing values were handled per predefined protocols to maintain data integrity and reliability.

### 4.2. Sociodemographic Characteristics and Grouping of the Patients

The following sociodemographic characteristics were recorded: age (calculated from the year of birth and year of surgery), body mass index (BMI; weight in kilograms divided by height in meters squared), smoking status, alcohol use, history of diabetes or hypertension, and any immunological disorders that could affect immune function. Cervical dysplasia data included results from routine screening and HPV testing, with a focus on high-risk HPV genotypes. Laboratory tests performed within one month before surgery were used to calculate inflammatory indices. SII and SIRI were derived using established formulas, with all hematologic parameters obtained from peripheral blood counts and expressed in giga/liter (G/L; 10^9^/L).

According to the Bethesda-based cervical cancer screening results and the histological findings of conizations, patients were categorized into four grades/groups: Grade 1 (G1) included negative findings, Grade 2 (G2) encompassed LSIL and ASC-US, Grade 3 (G3) comprised HSIL, ASC-H, AGC, and AIS, while Grade 4 (G4) represented cancer [35].

### 4.3. Statistical Analysis

Statistical analysis and figures were generated using GraphPad Prism 7.0 (GraphPad Software, San Diego, CA, USA) and IBM SPSS Statistics (version 25.0, IBM Corp. Armonk, NY, USA). Descriptive statistics were calculated to summarize the study population. Normality was assessed using the Kolmogorov–Smirnov, D’Agostino–Pearson omnibus, and Shapiro–Wilk tests. For normally distributed variables, unpaired Student’s *t*-tests were applied, while non-normally distributed data were analyzed using the Mann–Whitney U test. For comparisons across multiple groups, parametric analysis of variance (ANOVA) with Bonferroni post hoc correction or non-parametric Kruskal–Wallis tests with Dunn’s post hoc analysis was used, as appropriate. Values with normal distribution are expressed as mean ± standard error of the mean (SEM), while data with non-normal distribution are presented as median [interquartile range, IQR]. A two-tailed *p*-value < 0.05 was considered statistically significant. Significance symbols are as follows: *: *p* < 0.05; **: *p* < 0.01; ***: *p* < 0.001.

To evaluate the diagnostic performance of SII and SIRI, receiver operating characteristic (ROC) analyses were performed using binary outcomes defined as invasive cancer (Grade IV) versus non-invasive lesions (Grades I–III), based on cytological and histological classifications, respectively. Diagnostic accuracy was assessed by calculating the area under the ROC curve (AUC). Optimal cut-off values were identified using the Youden index and the Closest Top Left method, and corresponding sensitivity, specificity, positive predictive value (PPV), and negative predictive value (NPV) were reported. To assess whether SII and SIRI were independently associated with invasive disease, multivariable logistic regression analyses were performed using histology-confirmed invasive cancer (Grade IV) as the dependent variable. Models were specified a priori using an enter method and included SII (or SIRI), age, and smoking status to minimize overfitting, given the limited number of invasive cancer cases. Adjusted odds ratios (ORs) with 95% confidence intervals (CIs) were reported. Model fit was evaluated using Cox & Snell and Nagelkerke R^2^ values. Missing data were minimal for variables included in multivariable analyses; however, hr-HPV status and information on inflammatory or autoimmune conditions were unavailable for a substantial proportion of patients and were therefore not included in adjusted models.

## Figures and Tables

**Table 1:** Basic sociodemographic characteristics of the population. Categorical parameters are presented as n (pieces) and as a percentage (%). Continuous data are presented as median (interquartile range/IQR). #: 5 patients had missing weight and height data, thus BMI was not calculable, here *n* = 339.

Characteristics (*n* = 344)	N Range or Percentage (%)
Total number of the population	344
Age	
Median age (years)	40 (33–47)
Minimum age (years)	23
Maximum age (years)	78
Weight (*n* = 339) #	
Median weight (kg)	63 (56–73)
Minimum weight (kg)	14
Maximum weight (kg)	128
Body Mass Index/BMI (*n* = 339) #	
Median BMI (kg/m^2^)	22.7 (20.7–26.5)
Minimum BMI (kg/m^2^)	4.96
Maximum BMI (kg/m^2^)	46.5
Smoking (*n* = 344)	
Smoking Yes	56 (16.3%)
Smoking No	282 (82%)
Smoking No Data	6 (1.7%)
Alcohol (*n* = 344)	
Alcohol Yes	1 (0.3%)
Alcohol No	335 (97.4%)
Alcohol No Data	8 (2.3%)
Distribution of cytological samples (*n* = 344)	
G1	8 (2.3%)
G2	66 (19.2%)
G3	252 (73.3%)
G4	18 (5.2%)
High-risk HPV positivity (*n* = 344)	
hr-HPV Positivity Yes	196 (57%)
hr-HPV Positivity No	8 (2.3%)
hr-HPV Positivity No Data	140 (40.7%)
Identified hr-HPV and their types among positive hr-HPV results (*n* = 196)	
Identified hr HPV	54 (27.5%)
One hr HPV	39 (72.2%)
Two hr-HPV in combination	10 (18.5%)
Three hr-HPV in combination	5 (9.3%)
SII & SIRI (*n* = 344 for both)	
Median SII (G/L)	516.5 (358.8–701.6)
Median SIRI (G/L)	0.879 (0.611–1.24)

## Data Availability

The data are not publicly available due to privacy and ethical restrictions, however, data are available only on request from the corresponding author.

## Supplementary Materials

The following supporting information can be downloaded at: https://www.mdpi.com/article/10.3390/ijms27010435/s1.

## Abbreviations

The following abbreviations are used in this manuscript:

HPV	Human Papilloma Virus
CIN	Cervical Intraepithelial Neoplasia
LSIL	Low-grade Squamous Intraepithelial Lesion
HSIL	High-grade Squamous Intraepithelial Lesion
LEEP	Loop Electrosurgical Excision Procedure
NLR	Neutrophil-to-Lymphocyte Ratio
PLR	Platelet-to-Lymphocyte Ratio
SII	Systemic Immune-Inflammation Index
SIRI	Systemic Inflammation Response Index/SIRI
ASC-US	Atypical Squamous Cells of Undetermined Significance
ASC-H	Atypical Squamous Cells, cannot exclude High-grade lesion
AGC	Atypical Glandular Cells
AIS	Adenocarcinoma In Situ
ANOVA	Analysis of Variance
SEM	Standard Error of Mean
IQR	Interquartile Range
CI	Confidence Interval
OR	Odds Ratio
ROC	Receiver Operating Characteristic
AUC	Area Under Curve
NPV	Negative Predictive Value
PPV	Positive Predictive Value
IL-6;-8;-10	Interleukin-6;-8;-10
TGF-β	Transforming Growth Factor Beta
TANs	Tumor-associated Neutrophils
TAM	Tumor-associated Monocytes
