# Supplementary material for: Systemic Inflammatory Indices—Systemic Immune-Inflammation Index (SII) and the Systemic Inflammation Response Index (SIRI)—As Potential Rule-Out Biomarkers for Invasive Cervical Carcinoma
Source: Int J Mol Sci. 2025 Dec 31;27(1):435. doi: 10.3390/ijms27010435 (PMC12786036; doi:10.3390/ijms27010435)
Supplement: Supplementary file 1 [file ijms-27-00435-s001.zip › ijms-4010806-supplementary.pdf]

Supplementary Material for the following entitled manuscript:

Systemic Inflammatory Indices—Systemic Immune-Inflammation Index (SII) and the Systemic Inflammation Response Index (SIRI)—As Potential Rule-Out Biomarkers for Invasive Cervical Carcinoma

Márton Keszthelyi[1,†], Réka Eszter Sziva[1,†,*], Zsófia Havrán[2], Verita Szabó[2], Noémi Kalas[2], Lotti Lőczi[1], Barbara Sebők[3], Petra Merkely[1], Nándor Ács[1], , Szabolcs Várbíró[3,4], Balázs Lintner[1], Richárd Tóth[1]

1 Department of Obstetrics and Gynecology, Semmelweis University, 1082, Üllői Road 78/A, Budapest, Hungary; sziva.reka@semmelweis.hu, keszthelyi.marton@semmelweis.hu, keszthelyi.lotti.lucia@semmelweis.hu ; merkely.petra@gmail.com ; acs.nandor@semmelweis.hu ; lintner.balazs.zoltan@semmelweis.hu ; toth.richard@semmelweis.hu

2 Faculty of Medicine, Semmelweis University, 1085, Üllői Road 26, Budapest, Hungary; zsofi.havran@gmail.com ; szabo.verita@gmail.com ; kalasnoemi@gmail.com

3 Workgroup of Research Management, Doctoral School, Semmelweis University, 1085, Üllői Road 26, Budapest, Hungary; sebok.barbara23@gmail.com ; varbiroszabolcs@gmail.com

4 Department of Obstetrics and Gynecology, University of Szeged, 6725, Semmelweis Street 1, Szeged, Hungary

$ These authors contributed equally to this work and are considered first authors

* Correspondence: sziva.reka@semmelweis.hu

1§. HPV types among the examined patients

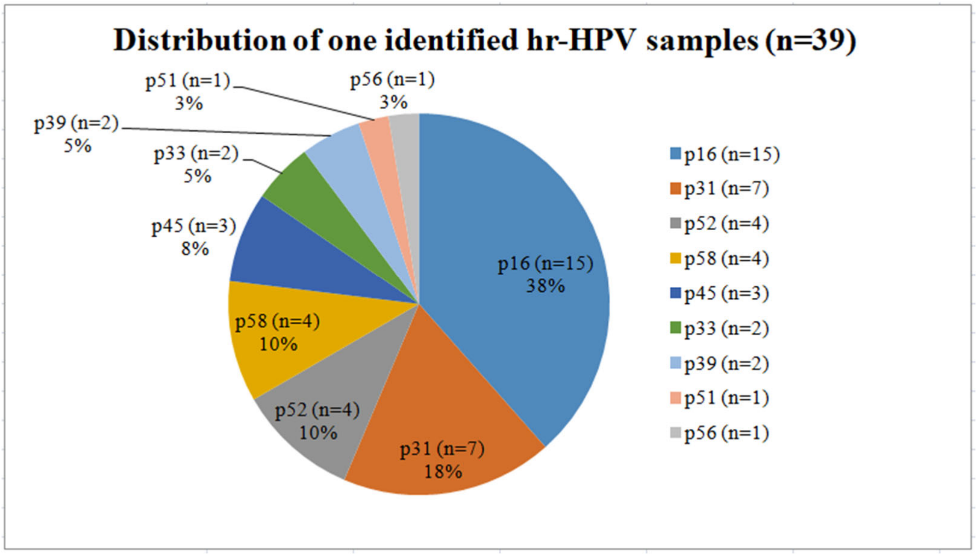

Diagram 1: Distribution of one identified hr-HPV samples. n=39.

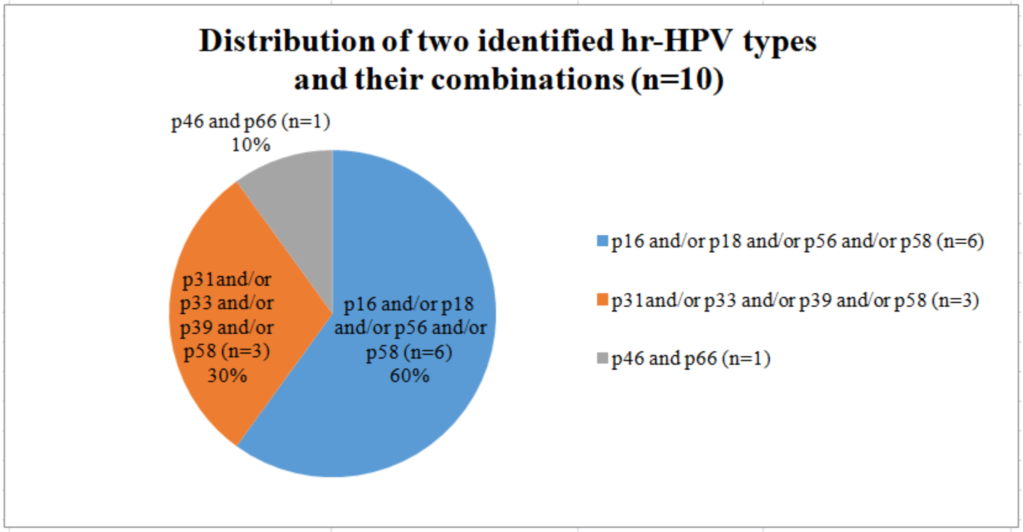

Diagram 2: Distribution of two identified hr-HPV types and their combinations. n=10.

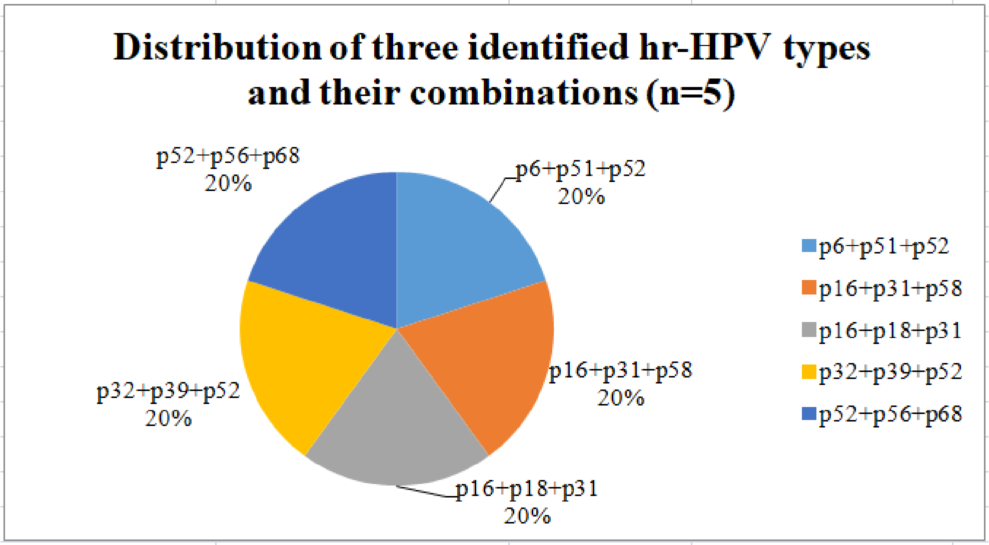

Diagram 3: Distribution of three identified hr-HPV types and their combinations. n=5.

2§. Descriptive statistics of Systemic Immune-Inflammation Index (SII) among the four groups. Grouping was based on the results of cervical cancer test screening.

Total: 344	Median SII	N	Interquartile range/IQR	Minimum	Maximum
Grade 1	764	8	246.8-1052	193.8	1928
Grade 2	447.9	66	310.3-609.0	147.2	1725
Grade 3	530.2	252	364.7-711.1	121.2	1962
Grade 4	564.1	18	428.7-773.5	299.8	1897

Table 1: SII values in the four groups by cervical cancer test screening results. Abbreviations: SII: Systemic Immune-inflammation Index, N: sample number in each group.

Comparison between groups	Mann-Whitney-U	P-value
Grade 1 vs. Grade 2	190.0	0.2007
Grade 1 vs. Grade 3	804.0	0.3311
Grade 1 vs. Grade 4	68.0	0.8458
Grade 2 vs. Grade 3	7058	0.0586
**Grade 2 vs. Grade 4**	**409.0**	**0.0443***
Grade 3 vs. Grade 4	1882	0.2284

Table 2: Mann-Whitney-U pairwise comparison of SII values between the four cytological groups. *: p<0.05 for G2 vs. G4.

3§. Descriptive statistics of System Inflammation Response Index (SIRI) among the four groups. Grouping was based on the results of cervical cancer test screening.

Total: 344	Median SIRI	N	Interquartile range/IQR	Minimum	Maximum
Grade 1	1.051	8	0.4907-1.656	0.3901	3.509
Grade 2	0.7977	66	0.5694-1.179	0.2200	3.294
Grade 3	0.8753	252	0.6044-1.248	0.0281	5.557
Grade 4	1.074	18	0.8354-1.380	0.6906	2.430

Table 3: SIRI values by cervical cancer test screening results. Abbreviations: SIRI: System Inflammation Response Index, N: sample number in each group.

Comparison between groups	Mann-Whitney-	P-value

	U	
Grade 1 vs. Grade 2	213.0	0.3794
Grade 1 vs. Grade 3	900.0	0.6077
Grade 1 vs. Grade 4	64.0	0.6769
Grade 2 vs. Grade 3	7702	0.3562
**Grade 2 vs. Grade 4**	**386.0**	**0.0237***
**Grade 3 vs. Grade 4**	**1621**	**0.0434***

Table 4: Mann-Whitney- U pairwise comparison of SIRI values between cytological groups. *: p<0.05 for G2 vs. G4 and G3 vs. G4.

4§. Descriptive statistics of Systemic Immune-Inflammation Index (SII) among the four groups. Grouping was based on the histological results of the LEEP-conization.

Total: 344	**Median SII**	**N**	**Interquartile range/IQR**	**Minimum**	**Maximum**
Grade 1	510.3	72	315.8-675.4	193.8	1928
Grade 2	448.0	30	356.4-582.9	161.8	1322
Grade 3	506.0	219	358.2-694.5	121.3	1962
Grade 4	767.5	23	550.0-1097	217.9	1897

Table 5: SII values in the four groups by histological results. Abbreviations: SII: Systemic Immune-inflammation Index, N: sample number in each group.

**Comparison between groups**	**Mann-Whitney-U**	**P-value**
Grade 1 vs. Grade 2	978.0	0.456
Grade 1 vs. Grade 3	7781	0.8686
**Grade 1 vs. Grade 4**	**437.0**	**0.0007*****
Grade 2 vs. Grade 3	2892	0.2887
**Grade 2 vs. Grade 4**	**152.0**	**0.0006*****
**Grade 3 vs. Grade 4**	**1352**	**0.0003*****

Table 6: Mann-Whitney-U pairwise comparison of SII values between histological groups. ***: p<0.001 for G1 vs G4, G2 vs. G4 and G3 vs. G4.

5§. Descriptive statistics of System Inflammation Response Index (SIRI) among the four groups. Grouping was based on the histological results of the LEEP-conization.

Total: 344	Median SIRI	N	Interquartile range/IQR	Minimum	Maximum
Grade 1	0.7657	72	0.5702-1.187	0.3367	3.509
Grade 2	0.8027	30	0.5702-1.162	0.2841	2.436
Grade 3	0.8770	219	0.6103-1.234	0.02811	4.279
Grade 4	1.32	23	0.9822-1.836	0.6802	5.557

Table 7: SIRI values by histological results. Abbreviations: SIRI: System Inflammation Response Index, N: sample number in each group.

Comparison between groups	Mann-Whitney-U	P-value
Grade 1 vs. Grade 2	1028	0.7053
Grade 1 vs. Grade 3	7327	0.3690
**Grade 1 vs. Grade 4**	**381.0**	**0.0001*** **
Grade 2 vs. Grade 3	2944	0.3574
**Grade 2 vs. Grade 4**	**142.0**	**0.0003*** **
**Grade 3 vs. Grade 4**	**1272**	**<0.0001*** **

Table 8: Mann-Whitney-U pairwise comparison of SIRI values between histological groups. ***: p<0.001 for G1 vs G4, G2 vs. G4 and G3 vs. G4.

6§. ROC-analysis results of Systemic Immune-Inflammation Index (SII) and System Inflammation Response Index (SIRI) and the results of cervical cancer test screening.

SII and cervical cancer test/cytology						
Cut-off Type	SII Cut-off	Sensitivity	Specificity	1 – Specificity	PPV	NPV
Youden-index	382.40	94.4%	32.2%	67.8%	7.1 %	99.1 %
Closest Top Left	461.34	72.2%	43.9%	56.1%	6.6 %	96.6 %
Area:	0.596625767					

Table 9: ROC analysis parameters for Systemic Immune Inflammation Index (SII) and cervical cancer test/cytology. Abbreviations: PPV=Positive Predictive Value, NPV=Negative Predictive Value.

SIRI and cervical cancer test/cytology						
Cut-off Type	SIRI Cut-off	Sensitivity	Specificity	1 – Specificity	PPV	NPV
Youden-index	0.691	100.0%	35.9%	64.1%	7.9%	100.0%
Closest Top Left	1.038	61.1%	63.2%	36.8%	8.4%	96.7%
Area:	0.647068848					

Table 10: ROC analysis parameters for System Inflammation Response Index (SIRI) and cervical cancer test/cytology. Abbreviations: PPV=Positive Predictive Value, NPV=Negative Predictive Value.

7§. ROC-analysis results of Systemic Immune-Inflammation Index (SII) and System Inflammation Response Index (SIRI) and the results of histological examination

SII and histology

Cut-off Type	SII Cut-off	Sensitivity	Specificity	1 – Specificity	PPV	NPV
Youden-index	681.74	65.2%	75.7%	24.3%	16.1%	96.8%
Closest Top Left	681.74	65.2%	75.7%	24.3%	16.1%	96.8%
Area:	0.73709874					

Table 11: ROC analysis parameters for Systemic Immune Inflammation Index (SII) and histology. Abbreviations: PPV=Positive Predictive Value, NPV=Negative Predictive Value.

SIRI and histology						
Cut-off Type	SIRI Cut-off	Sensitivity	Specificity	1 – Specificity	PPV	NPV
Youden-index	0.915	87.0%	55.5%	44.6%	12.3%	98.3%
Closest Top Left	1.037	73.9%	64.2%	35.8%	12.9%	97.2%
Area:	0.756873899					

Table 12. ROC analysis parameters for System Inflammation Response Index (SIRI) and histology. Abbreviations: PPV=Positive Predictive Value, NPV=Negative Predictive Value.

<u>8§. Multivariable Logistic Regression – Original data</u>

Logistic Regression

Hystology & SII és Age, BMI, Smoking

/Dohányzás

Case Processing Summary

Unweighted Cases[a]		N	Percent
Selected Cases	Included in Analysis	335	97,4
	Missing Cases	9	2,6
	Total	344	100,0
Unselected Cases		0	0,0
Total		344	100,0

a. If weight is in effect, see classification table for the total number of cases.

Dependent Variable Encoding

Original Value	Internal Value
0	0
1	1

Block 0: Beginning Block

Classification Table[a,b]

Observed			Predicted: Hisztológiai eredmény alapján rák-e 0	1	Percentage Correct
Step 0	Hisztológiai eredmény alapján rák-e	0	312	0	100,0
		1	23	0	0,0
	Overall Percentage				93,1

a. Constant is included in the model.

b. The cut value is ,500

Variables in the Equation

		B	S.E.	Wald	df	Sig.	Exp(B)
Step 0	Constant	-2,608	0,216	145,643	1	0,000	0,074

Variables not in the Equation

			Score	df	Sig.
Step 0	Variables	SII	22,392	1	0,000
		Dohányzás	0,002	1	0,960
		BMI	0,219	1	0,640

Age	9,115	1	0,003
Overall Statistics	30,360	4	0,000

Block 1: Method = Enter

Omnibus Tests of Model Coefficients

		Chi-square	df	Sig.
Step 1	Step	24,994	4	0,000
	Block	24,994	4	0,000
	Model	24,994	4	0,000

Model Summary

Step	-2 Log likelihood	Cox & Snell R Square	Nagelkerke R Square
1	142,606[a]	0,072	0,183

a. Estimation terminated at iteration number 6 because parameter estimates changed by less than ,001.

Classification Table[a]

Observed			Predicted: Hisztológiai eredmény alapján rák-e 0	Predicted: Hisztológiai eredmény alapján rák-e 1	Percentage Correct
Step 1	Hisztológiai eredmény alapján rák-e	0	310	2	99,4
		1	22	1	4,3
	Overall Percentage				92,8

a. The cut value is ,500

Variables in the Equation

		B	S.E.	Wald	df	Sig.	Exp(B)
Step 1[a]	SII	0,002	0,001	17,510	1	0,000	1,002
	Dohányzás	-0,066	0,608	0,012	1	0,913	0,936
	BMI	-0,040	0,047	0,695	1	0,404	0,961
	Age	0,066	0,023	8,571	1	0,003	1,068
	Constant	-6,069	1,366	19,729	1	0,000	0,002

a. Variable(s) entered on step 1: SII, Dohányzás, BMI, Age.

Logistic Regression

Case Processing Summary

Unweighted Cases[a]	N	Percent

Selected Cases	Included in Analysis	338	98,3
	Missing Cases	6	1,7
	Total	344	100,0
Unselected Cases		0	0,0
Total		344	100,0

a. If weight is in effect, see classification table for the total number of cases.

Dependent Variable Encoding

Original Value	Internal Value
0	0
1	1

Block 0: Beginning Block

Classification Table[a,b]

Observed			Predicted: Hisztológiai eredmény alapján rák-e 0	1	Percentage Correct
Step 0	Hisztológiai eredmény alapján rák-e	0	315	0	100,0
		1	23	0	0,0
	Overall Percentage				93,2

a. Constant is included in the model.

b. The cut value is ,500

Variables in the Equation

		B	S.E.	Wald	df	Sig.	Exp(B)
Step 0	Constant	-2,617	0,216	146,810	1	0,000	0,073

Variables not in the Equation

			Score	df	Sig.
Step 0	Variables	SII	22,458	1	0,000
		Dohányzás	0,005	1	0,944
		Age	8,914	1	0,003
	Overall Statistics		28,993	3	0,000

Block 1: Method = Enter

Omnibus Tests of Model Coefficients

		Chi-square	df	Sig.
Step 1	Step	24,130	3	0,000
	Block	24,130	3	0,000
	Model	24,130	3	0,000

Model Summary

Step	-2 Log likelihood	Cox & Snell R Square	Nagelkerke R Square
1	143,895[a]	0,069	0,176

a. Estimation terminated at iteration number 6 because parameter estimates changed by less than ,001.

Classification Table[a]

Observed			Predicted: Hisztológiai eredmény alapján rák-e 0	1	Percentage Correct
Step 1	Hisztológiai eredmény alapján rák-e	0	313	2	99,4
		1	22	1	4,3
	Overall Percentage				92,9

a. The cut value is ,500

Variables in the Equation

		B	S.E.	Wald	df	Sig.	Exp(B)
Step 1[a]	SII	0,002	0,001	16,988	1	0,000	1,002
	Dohányzás	0,001	0,601	0,000	1	0,998	1,001
	Age	0,059	0,021	7,813	1	0,005	1,061
	Constant	-6,737	1,129	35,588	1	0,000	0,001

a. Variable(s) entered on step 1: SII, Dohányzás, Age.

Logistic Regression

Case Processing Summary

Unweighted Cases[a]		N	Percent
Selected Cases	Included in Analysis	335	97,4
	Missing Cases	9	2,6
	Total	344	100,0
Unselected Cases		0	0,0
Total		344	100,0

a. If weight is in effect, see classification table for

the total number of cases.

Dependent Variable Encoding

Original Value	Internal Value
0	0
1	1

Block 0: Beginning Block

Classification Table[a,b]

Observed			Predicted: Hisztológiai eredmény alapján rák-e 0	Predicted: Hisztológiai eredmény alapján rák-e 1	Percentage Correct
Step 0	Hisztológiai eredmény alapján rák-e	0	312	0	100,0
		1	23	0	0,0
	Overall Percentage				93,1

a. Constant is included in the model.

b. The cut value is ,500

Variables in the Equation

		B	S.E.	Wald	df	Sig.	Exp(B)
Step 0	Constant	-2,608	0,216	145,643	1	0,000	0,074

Variables not in the Equation

			Score	df	Sig.
Step 0	Variables	SIRI	16,692	1	0,000
		Dohányzás	0,002	1	0,960
		Age	9,115	1	0,003
		BMI	0,219	1	0,640
	Overall Statistics		24,827	4	0,000

Block 1: Method = Enter

Omnibus Tests of Model Coefficients

		Chi-square	df	Sig.
Step 1	Step	19,339	4	0,001
	Block	19,339	4	0,001
	Model	19,339	4	0,001

Model Summary

Step	-2 Log likelihood	Cox & Snell R Square	Nagelkerke R Square
1	148,261[a]	0,056	0,142

a. Estimation terminated at iteration number 6 because parameter estimates changed by less than ,001.

Classification Table[a]

Observed			Predicted: Hisztológiai eredmény alapján rák-e 0	Predicted: Hisztológiai eredmény alapján rák-e 1	Percentage Correct
Step 1	Hisztológiai eredmény alapján rák-e	0	310	2	99,4
		1	22	1	4,3
	Overall Percentage				92,8

a. The cut value is ,500

Variables in the Equation

		B	S.E.	Wald	df	Sig.	Exp(B)
Step 1[a]	SIRI	0,831	0,238	12,138	1	0,000	2,295
	Dohányzás	-0,239	0,628	0,145	1	0,703	0,787
	Age	0,060	0,021	7,857	1	0,005	1,062
	BMI	-0,021	0,044	0,217	1	0,641	0,980
	Constant	-5,679	1,266	20,109	1	0,000	0,003

a. Variable(s) entered on step 1: SIRI, Dohányzás, Age, BMI.

```
LOGISTIC REGRESSION VARIABLES hyst_gradeIV
  /METHOD=ENTER SIRI cigi Age
  /CRITERIA=PIN(.05) POUT(.10) ITERATE(20) CUT(.5).
```

Logistic Regression

Case Processing Summary

Unweighted Cases[a]		N	Percent
Selected Cases	Included in Analysis	338	98,3
	Missing Cases	6	1,7
	Total	344	100,0
Unselected Cases		0	0,0
Total		344	100,0

a. If weight is in effect, see classification table for the total number of cases.

Dependent

Variable Encoding

Original Value	Internal Value
0	0
1	1

Block 0: Beginning Block

Classification Table[a,b]

Observed			Predicted: Hisztológiai eredmény alapján rák-e 0	1	Percentage Correct
Step 0	Hisztológiai eredmény alapján rák-e	0	315	0	100,0
		1	23	0	0,0
	Overall Percentage				93,2

a. Constant is included in the model.

b. The cut value is ,500

Variables in the Equation

		B	S.E.	Wald	df	Sig.	Exp(B)
Step 0	Constant	-2,617	0,216	146,810	1	0,000	0,073

Variables not in the Equation

			Score	df	Sig.
Step 0	Variables	SIRI	16,818	1	0,000
		Dohányzás	0,005	1	0,944
		Age	8,914	1	0,003
	Overall Statistics		24,255	3	0,000

Block 1: Method = Enter

Omnibus Tests of Model Coefficients

		Chi-square	df	Sig.
Step 1	Step	19,019	3	0,000
	Block	19,019	3	0,000
	Model	19,019	3	0,000

Model Summary

Step	-2 Log likelihood	Cox & Snell R Square	Nagelkerke R Square
1	149,006[a]	0,055	0,140

a. Estimation terminated at iteration number 6 because parameter estimates changed by less than ,001.

Classification Table[a]

Observed			Predicted: Hisztológiai eredmény alapján rák-e 0	1	Percentage Correct
Step 1	Hisztológiai eredmény alapján rák-e	0	314	1	99,7
		1	22	1	4,3
	Overall Percentage				93,2

a. The cut value is ,500

Variables in the Equation

		B	S.E.	Wald	df	Sig.	Exp(B)
Step 1[a]	SIRI	0,837	0,239	12,284	1	0,000	2,308
	Dohányzás	-0,200	0,624	0,103	1	0,748	0,818
	Age	0,056	0,020	7,825	1	0,005	1,058
	Constant	-6,039	1,017	35,269	1	0,000	0,002

a. Variable(s) entered on step 1: SIRI, Dohányzás, Age.

```
LOGISTIC REGRESSION VARIABLES cyst_gradeIV
  /METHOD=ENTER SII cigi Age BMI
  /CRITERIA=PIN(.05) POUT(.10) ITERATE(20) CUT(.5).
```

Logistic Regression

Case Processing Summary

Unweighted Cases[a]		N	Percent
Selected Cases	Included in Analysis	335	97,4
	Missing Cases	9	2,6
	Total	344	100,0
Unselected Cases		0	0,0
Total		344	100,0

a. If weight is in effect, see classification table for the total number of cases.

Dependent Variable Encoding

Original Value	Internal Value
0	0

1	1

Block 0: Beginning Block

Classification Table[a,b]

Observed			Predicted: Citológiai eredmény alapján rák-e 0	1	Percentage Correct
Step 0	Citológiai eredmény alapján rák-e	0	317	0	100,0
		1	18	0	0,0
	Overall Percentage				94,6

a. Constant is included in the model.

b. The cut value is ,500

Variables in the Equation

		B	S.E.	Wald	df	Sig.	Exp(B)
Step 0	Constant	-2,869	0,242	140,154	1	0,000	0,057

Variables not in the Equation

			Score	df	Sig.
Step 0	Variables	SII	5,302	1	0,021
		Dohányzás	0,470	1	0,493
		Age	10,944	1	0,001
		BMI	4,327	1	0,038
	Overall Statistics		16,235	4	0,003

Block 1: Method = Enter

Omnibus Tests of Model Coefficients

		Chi-square	df	Sig.
Step 1	Step	14,503	4	0,006
	Block	14,503	4	0,006
	Model	14,503	4	0,006

Model Summary

Step	-2 Log likelihood	Cox & Snell R Square	Nagelkerke R Square
1	125,768[a]	0,042	0,124

a. Estimation terminated at iteration number 6 because parameter estimates changed by less than ,001.

Classification Table[a]

Observed			Predicted: Citológiai eredmény alapján rák-e 0	1	Percentage Correct
Step 1	Citológiai eredmény alapján rák-e	0	317	0	100,0
		1	18	0	0,0
	Overall Percentage				94,6

a. The cut value is ,500

Variables in the Equation

		B	S.E.	Wald	df	Sig.	Exp(B)
Step 1[a]	SII	0,001	0,001	4,053	1	0,044	1,001
	Dohányzás	-0,587	0,786	0,557	1	0,455	0,556
	Age	0,063	0,023	7,439	1	0,006	1,065
	BMI	0,034	0,043	0,642	1	0,423	1,035
	Constant	-7,226	1,402	26,569	1	0,000	0,001

a. Variable(s) entered on step 1: SII, Dohányzás, Age, BMI.

```
LOGISTIC REGRESSION VARIABLES cyst_gradeIV
  /METHOD=ENTER SII cigi Age
  /CRITERIA=PIN(.05) POUT(.10) ITERATE(20) CUT(.5).
```

Logistic Regression

Notes

Case Processing Summary

Unweighted Cases[a]		N	Percent
Selected Cases	Included in Analysis	338	98,3
	Missing Cases	6	1,7
	Total	344	100,0
Unselected Cases		0	0,0
Total		344	100,0

a. If weight is in effect, see classification table for the total number of cases.

Dependent Variable Encoding

Original Value	Internal Value
0	0
1	1

Block 0: Beginning Block

Classification Table[a,b]

Observed			Predicted: Citológiai eredmény alapján rák-e 0	1	Percentage Correct
Step 0	Citológiai eredmény alapján rák-e	0	320	0	100,0
		1	18	0	0,0
	Overall Percentage				94,7

a. Constant is included in the model.

b. The cut value is ,500

Variables in the Equation

		B	S.E.	Wald	df	Sig.	Exp(B)
Step 0	Constant	-2,878	0,242	141,147	1	0,000	0,056

Variables not in the Equation

			Score	df	Sig.
Step 0	Variables	SII	5,299	1	0,021
		Dohányzás	0,449	1	0,503
		Age	10,755	1	0,001
	Overall Statistics		15,240	3	0,002

Block 1: Method = Enter

Omnibus Tests of Model Coefficients

		Chi-square	df	Sig.
Step 1	Step	13,725	3	0,003
	Block	13,725	3	0,003
	Model	13,725	3	0,003

Model Summary

Step	-2 Log likelihood	Cox & Snell R Square	Nagelkerke R Square
1	126,876[a]	0,040	0,117

a. Estimation terminated at iteration number 6 because parameter estimates changed by less than ,001.

Classification Table[a]

Observed	Predicted: Citológiai eredmény alapján rák-e	Percentage Correct

			0	1	
Step 1	Citológiai eredmény alapján rák-e	0	320	0	100,0
		1	18	0	0,0
	Overall Percentage				94,7

a. The cut value is ,500

Variables in the Equation

		B	S.E.	Wald	df	Sig.	Exp(B)
Step 1[a]	SII	0,001	0,001	4,537	1	0,033	1,001
	Dohányzás	-0,609	0,784	0,603	1	0,437	0,544
	Age	0,068	0,022	9,565	1	0,002	1,071
	Constant	-6,646	1,181	31,643	1	0,000	0,001

a. Variable(s) entered on step 1: SII, Dohányzás, Age.

```
LOGISTIC REGRESSION VARIABLES cyst_gradeIV
  /METHOD=ENTER SIRI cigi Age BMI
  /CRITERIA=PIN(.05) POUT(.10) ITERATE(20) CUT(.5).
```

Logistic Regression

Case Processing Summary

Unweighted Cases[a]		N	Percent
Selected Cases	Included in Analysis	335	97,4
	Missing Cases	9	2,6
	Total	344	100,0
Unselected Cases		0	0,0
Total		344	100,0

a. If weight is in effect, see classification table for the total number of cases.

Dependent Variable Encoding

Original Value	Internal Value
0	0
1	1

Block 0: Beginning Block

Classification Table[a,b]

Observed			Predicted Citológiai eredmény alapján rák-e 0	1	Percentage Correct
Step 0	Citológiai eredmény alapján rák-e	0	317	0	100,0
		1	18	0	0,0
	Overall Percentage				94,6

a. Constant is included in the model.

b. The cut value is ,500

Variables in the Equation

		B	S.E.	Wald	df	Sig.	Exp(B)
Step 0	Constant	-2,869	0,242	140,154	1	0,000	0,057

Variables not in the Equation

			Score	df	Sig.
Step 0	Variables	SIRI	1,334	1	0,248
		Dohányzás	0,470	1	0,493
		Age	10,944	1	0,001
		BMI	4,327	1	0,038
	Overall Statistics		13,556	4	0,009

Block 1: Method = Enter

Omnibus Tests of Model Coefficients

		Chi-square	df	Sig.
Step 1	Step	11,763	4	0,019
	Block	11,763	4	0,019
	Model	11,763	4	0,019

Model Summary

Step	-2 Log likelihood	Cox & Snell R Square	Nagelkerke R Square
1	128,507[a]	0,035	0,101

a. Estimation terminated at iteration number 6 because parameter estimates changed by less than ,001.

Classification Table[a]

Observed			Predicted Citológiai eredmény alapján rák-e 0	1	Percentage Correct
Step 1	Citológiai eredmény alapján	0	317	0	100,0
		1	18	0	0,0

	rák-e			
	Overall Percentage			94,6

a. The cut value is ,500

Variables in the Equation

		B	S.E.	Wald	df	Sig.	Exp(B)
Step 1[a]	SIRI	0,311	0,323	0,929	1	0,335	1,365
	Dohányzás	-0,619	0,787	0,619	1	0,432	0,538
	Age	0,060	0,023	7,191	1	0,007	1,062
	BMI	0,040	0,041	0,946	1	0,331	1,041
	Constant	-6,777	1,326	26,121	1	0,000	0,001

a. Variable(s) entered on step 1: SIRI, Dohányzás, Age, BMI.

```
LOGISTIC REGRESSION VARIABLES cyst_gradeIV
  /METHOD=ENTER SIRI cigi Age
  /CRITERIA=PIN(.05) POUT(.10) ITERATE(20) CUT(.5).
```

Logistic Regression

Case Processing Summary

Unweighted Cases[a]		N	Percent
Selected Cases	Included in Analysis	338	98,3
	Missing Cases	6	1,7
	Total	344	100,0
Unselected Cases		0	0,0
Total		344	100,0

a. If weight is in effect, see classification table for the total number of cases.

Dependent Variable Encoding

Original Value	Internal Value
0	0
1	1

Block 0: Beginning Block

Classification Table[a,b]

Observed		Predicted	
		Citológiai eredmény alapján rák-e	Percentage Correct

			0	1	
Step 0	Citológiai eredmény alapján rák-e	0	320	0	100,0
		1	18	0	0,0
	Overall Percentage				94,7

a. Constant is included in the model.

b. The cut value is ,500

Variables in the Equation

		B	S.E.	Wald	df	Sig.	Exp(B)
Step 0	Constant	-2,878	0,242	141,147	1	0,000	0,056

Variables not in the Equation

			Score	df	Sig.
Step 0	Variables	SIRI	1,345	1	0,246
		Dohányzás	0,449	1	0,503
		Age	10,755	1	0,001
	Overall Statistics		12,138	3	0,007

Block 1: Method = Enter

Omnibus Tests of Model Coefficients

		Chi-square	df	Sig.
Step 1	Step	10,721	3	0,013
	Block	10,721	3	0,013
	Model	10,721	3	0,013

Model Summary

Step	-2 Log likelihood	Cox & Snell R Square	Nagelkerke R Square
1	129,879[a]	0,031	0,092

a. Estimation terminated at iteration number 6 because parameter estimates changed by less than ,001.

Classification Table[a]

Observed			Predicted: Citológiai eredmény alapján rák-e 0	Predicted: Citológiai eredmény alapján rák-e 1	Percentage Correct
Step 1	Citológiai eredmény alapján rák-e	0	320	0	100,0
		1	18	0	0,0
	Overall Percentage				94,7

a. The cut value is ,500

Variables in the Equation

		B	S.E.	Wald	df	Sig.	Exp(B)
Step 1[a]	SIRI	0,331	0,311	1,137	1	0,286	1,393
	Dohányzás	-0,650	0,787	0,681	1	0,409	0,522
	Age	0,067	0,022	9,610	1	0,002	1,069
	Constant	-6,075	1,096	30,745	1	0,000	0,002

a. Variable(s) entered on step 1: SIRI, Dohányzás, Age.
